# The effect of the demonstration-based progressive muscle relaxation technique on stress and anxiety in nurses caring for COVID-19 patients: a randomized clinical trial

**DOI:** 10.1186/s12888-022-04456-3

**Published:** 2022-12-15

**Authors:** Samaneh Ganjeali, Zahra Farsi, Seyedeh Azam Sajadi, Kourosh Zarea

**Affiliations:** 1 Student Research Committee Department, Faculty of Nursing, Aja University of Medical Sciences, Tehran, Iran; 2 Research and Community Health Departments, Faculty of Nursing, Aja University of Medical Sciences, Kaj St., Shariati St, Tehran, Iran; 3 Nursing Management Department, Faculty of Nursing, Aja University of Medical Sciences, Tehran, Iran; 4 Nursing Care Research Center in Chronic Diseases. School of Nursing and Midwifery, Ahvaz Jundishapur University of Medical Sciences, Ahvaz, Iran

**Keywords:** Relaxation, Muscle Relaxation, Stress, Anxiety, COVID-19, Nurses, Education, Training

## Abstract

**Background:**

Caring for patients with coronavirus disease 2019 (COVID-19) challenges nurses and causes them to experience stress and anxiety. From this perspective, it is of utmost importance to develop quick and effective intervention strategies to prevent numerous complications. This study aimed to investigate the effect of the progressive muscle relaxation technique, using the demonstration method on the stress and anxiety of nurses who care for COVID-19 patients.

**Methods:**

This randomized clinical trial was conducted in 2021. Forty-six nurses working in two referral hospitals with wards for COVID-19 patients in Tehran, Iran recruited by convenience sampling method and then randomly assigned to experimental or control groups. The nurses in the experimental group educated the progressive muscle relaxation by the demonstration method, and they were encouraged to practice it. The Depression Anxiety Stress Scale-21 was utilized to measure the stress and anxiety levels in the nurses.

**Results:**

Before the intervention, the levels of stress in the experimental and control groups were 13.91 ± 2.41 vs. 14.34 ± 2.74 (*p* = 0.571), and their anxiety was 13.34 ± 3.41 vs. 12.78 ± 2.21 (*p* = 0.510), respectively. After the intervention, the levels of stress in the experimental and control groups were 10.95 ± 2.01 vs. 14.17 ± 2.34 (*p* < 0.001), and their anxiety was 9.47 ± 2.37 vs. 12.91 ± 1.85 (*p* < 0.001), respectively. Moreover, the levels of stress and anxiety in the experimental group significantly diminished after intervention (*p* < 0.001), but no significant changes were observed in the control group (*p* > 0.05).

**Conclusion:**

Concerning the effectiveness of the progressive muscle relaxation technique in relieving the stress and anxiety of the nurses caring for COVID-19 patients, it is suggested to include this relaxation technique in nursing courses.

## Background

Since March 11, 2020, when the World Health Organization declared the novel coronavirus disease 2019 (COVID-19) a pandemic has been focused more on global health [1]. The COVID-19 outbreak has created a shock in the healthcare systems in most countries, including Iran [2]. Caring for patients with COVID-19 has further given rise to countless psychological complications, including stress and anxiety in nurses [3–6]. For instance, a study reported that the prevalence rates of stress and anxiety among nurses who provided care to patients with COVID-19 were 54.9% and 89.7%, respectively [5]. Also, evidence showed that the stress and anxiety of Iranian nurses increased significantly first wave of the COVID-19 pandemic [6]. A recent study showed that caregiving stress, impression on all aspects of life, COVID as a strange disease, stress caused by patient characteristics, and stress reduction over time were issues in the formation of stress in nurses caring for patients with COVID-19 [7].

The primary source of stress is known and an external factor often induce it. While multiple unpleasant emotions and hypothetical experiences cause anxiety [8]. Anxiety has been further introduced as one of the negative impacts of stress. Thus, stress can develop into anxiety as individuals find themselves in stressful situations and are even unable to control them. As well, stress can be decreased after coping with stressful events, but anxiety becomes a life routine and persists for a long time [8].

Since the COVID-19 pandemic started, nurses have been on the frontline of fighting against this disease, caring for infected patients, and have primarily suffered from work pressure. Their performance has been further disturbed by some issues, such as the inadequate supply of personal protective equipment in many healthcare facilities, media pressure, and no support from relevant authorities [9–11]. In critical situations, such as the COVID-19 pandemic, nurses always play against a mixture of circumstances with limited resources to care for patients, so they are often suffered high levels of stress [12, 13]. For example, Mohamadzadeh Tabrizi et al. reported that half of the nurses working in the wards for COVID-19 cases had experienced moderate-to-severe anxiety [14]. Ariapooran et al. also found that more than 51% of nurses working in the covid-19 ward suffered from secondary stress disorder caused by covid-19 [15]. As well, de Pinho et al. revealed that 64% of frontline nurses fighting against COVID-19 in Portugal were experiencing abnormal stress [16].

Exposure to COVID-19 can also have double effects on mental health, so one of the complications of contracting this virus is its mental consequences, on the one hand, patients with mental illnesses are susceptible to this pandemic, on the other hand [17]. Evidence has further demonstrated that the unknown nature of COVID-19, insufficient knowledge about the novel disease, mainly at its onset, patients' conditions, no definitive medications, rapid viral mutations, fear of being infected or losing one's life, transmitting the disease to family and friends, no support from relevant authorities and organizations, high treatment costs in case of infection, elevated workload, and worsened nursing shortage cause to increase stress and anxiety in nurses [18, 19]. According to Doo et al., the incidence rates of anxiety among the nurses working in the COVID-19 wards were significantly higher versus those involved in other wards [20].

Since nurses' anxiety cause decreased efficiency, and increase mental and physical injuries as well as dissatisfaction with healthcare services [21], selecting the best solution can reduce the negative impacts on their mental health and quality of care. So far, various intervention strategies have been implemented in this field, including the progressive muscle relaxation (PMR) technique [22]. Relaxation here refers to the establishment of a specific state of moderation, which is the opposite of tensions, such as anxiety [23]. First proposed by Jacobson, PMR is a technique that affects physiology by slowing the body's metabolism and increasing the number and strength of heart contractions, respiration rates, epinephrine secretion levels, and blood pressure, and aiding people in promoting their physical and mental health [24]. In this line, Özlü et al. reported that the relaxation technique could relieve anxiety in COVID-19 patients [25]. Also, Liu et al. found that PMR can reduce anxiety in patients with COVID-19 [26]. Toqan et al. indicated the positive influence of PMR on nursing students’ anxiety levels in pediatric clinical settings [27]. On the other hand, Matourypour et al. found that implementing this technique as an emotion-oriented adaptation strategy could not diminish the psychological problems facing nurses [28]. We found that most of the studies have examined the effectiveness of PMR on patients and we did not find any study that assessed its effects on stress and anxiety nurses during the COVID pandemic. Given the contradictory results in the previous studies, this study aimed to investigate the effectiveness of PMR using the demonstration method on stress and anxiety in nurses caring for COVID-19 patients.

## Methods

### Design

This randomized clinical trial was conducted from March to September 2021 and it was registered on the Iranian Registry of Clinical Trials (ID: IRCT20210808052111N1, Registration date: 04/09/2021). The entire trial protocol can be accessed at Aja University of Medical Sciences.

### Participants and setting

The statistical population consisted of all nurses working in two referral hospitals with wards for COVID-19 patients in Tehran, Iran. Of note, both hospitals were comparable in terms of workload, organizational culture, and nursing staff. The sample size was also estimated to be about 19 nurses in each group, according to the sample size formula, the mean and standard deviation (SD) reported in the previous study [29], with a 99% confidence interval, and a 95% test power. Considering the possibility of 20% attrition, 23 nurses in each group and 46 individuals in total were recruited by convenience sampling. The hospitals were further divided into two experimental and control groups through simple random sampling by coin flipping. A researcher assistant generated the random allocation sequence.

$$n= \frac{{\left({\mathrm{Z}}_{1-\frac{\alpha }{2}}+{\mathrm{Z}}_{1-\beta }\right)}^{2}(\delta {1}^{2}+\delta {2}^{2})}{{\left({\upmu }_{1}-{\upmu }_{2}\right)}^{2}}=\frac{{\left(2.58+1.64\right)}^{2}\left({\left(4.98\right)}^{2}+{\left(8.77\right)}^{2}\right)}{{\left(11.38\right)}^{2}}= 19.32$$

The inclusion criteria were at least a Bachelor's degree in nursing, at least six months of clinical work experience, taking no anxiolytic and psychoactive medications, not receiving any training via the Jacobson's PMR, and experiencing no critical situations (such as the death of the beloved ones and/or incurable diseases) over the last six months. The exclusion criteria were withdrawal from the study, absenteeism even for one training session, being infected with COVID-19, having first-degree relatives with COVID-19 confirmed, and undergoing stressful events at the time of the study. One of the researchers enrolled participants and assigned them to intervention and control groups.

### Data collection

The data collection tool consisted of the demographic characteristics questionnaire (gender, age, marital status, education, number of children, clinical work experience, hospital, ward, and work shift) and the 21-item Depression Anxiety Stress Scale-21 (DASS-21), designed by Lovibond and Lovibond in 1995. This questionnaire comprised three 7-item subscales, and the final score for each one was obtained through the sum of the values of the related items. Each item was further scored from 0 (*does not apply to me at all*) to 3 (*totally applies to me*). As DASS-21 was the short form of the main scale (with 42 items), the final score needed to be doubled for each subscale. The researchers used the stress and anxiety subscales in this article. In 1995, Lovibond and Lovibond confirmed the validity of this questionnaire as 0.77. Also, they confirmed the reliability of the entire questionnaire and its subscales with Cronbach's alpha coefficient [30]. In addition, Taheri Gharagzlu et al. found the Cronbach's alpha coefficients for the depression, anxiety, and stress subscales to be 97%, 92%, and 95%, respectively, and the test–retest coefficients for these dimensions were 79%, 67%, and 81%, respectively [31]. Both experimental and control groups completed the DASS-21 questionnaire in the pre-and post-intervention stages.

### Intervention

First, one of the researchers (viz. a Master's student of Military Nursing with ten years of clinical work experience in general, critical care unit, and COVID-19 wards) referred to certified psychiatrists and clinical psychologists, and learned about PMR, acquired the necessary skills, received an approval certificate in this field. Then she implemented the PMR, with much focus on four components (a quiet environment, a mental tool like a word for concentration, a passive attitude, and a comfortable position) to reduce stress and anxiety in the nurses [29]. Then, the researcher taught PMR to the nurses in the experimental group, divided into groups of six nurses, in a quiet environment (here, the hospital venue) for two 60-min sessions every other day. During the first session, the nurses were trained in PMR by demonstration. In the second session, the nurses practiced the PMR to consolidate the training. All the sessions were videotaped and provided to the experimental group through a compact disc. Then, the nurses were encouraged to practice the PMR.

**Fig. 1:**
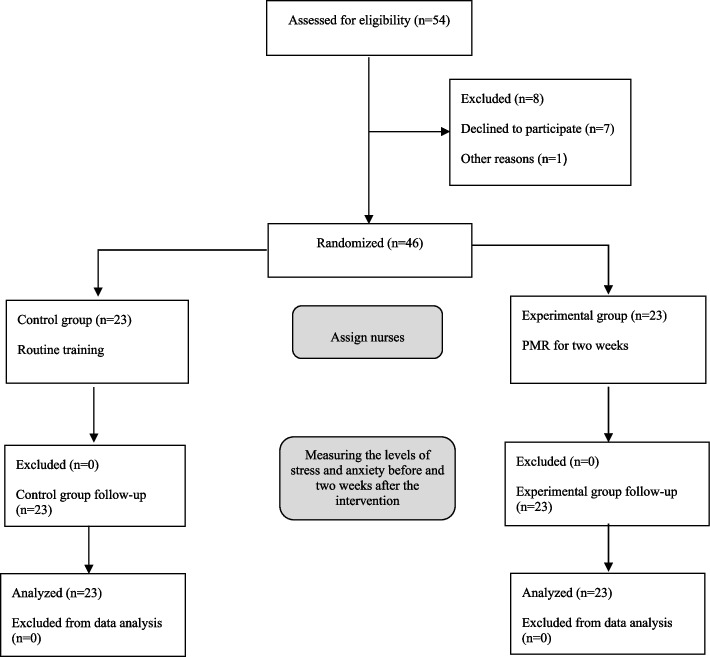
The process of study

For this purpose, the nurses could sit or lie in a comfortable chair. Then, they put on loose clothing if possible and took off their watch, bracelets, and other accessories. A piece of light music was subsequently played during the intervention. Afterward, the nurses tensed and relaxed their muscles with effective and deep breathing while listening to the audio file. Such exercises were more tangible for the nurses at the start. Once they tensed and relaxed their muscles, they could chill out and release their whole body easily and spontaneously with PMR, and divested themselves of anxiety, stress, and any other unpleasant mental and physical feelings [32]. The muscles that are mostly used in PMR exercise include “hand and forearm, forehead, upper arm, mouth and jaw, eyes and cheeks, shoulder blades, shoulders, neck, chest and stomach, hips and buttocks, upper leg, lower leg, and foot”. These muscles should be contracted and expanded in the order of Table 1 [27]. Next, the nurses were requested to practice this technique once a day for two weeks, each time lasting 20 min, in a quiet environment and a comfortable position. Of note, they were encouraged to do so via a phone call every day. After two weeks, the researchers assessed the stress and anxiety of nurses in the experimental group. However, the control group received no training or intervention. They completed the stress and anxiety subscales of DASS-21 after two weeks. No sample loss occurred in this study (Fig. 1).

**Table 1:** Progressive muscle relaxation (PMR) exercise

**Part of body**	**Exercise**
Hand and forearm	“Clench your hand into a fist”
Forehead	“Rise your eyebrows as much as you can, as if you were startled or shocked”
Upper arm	“Rise your right forearm and flex your bicep,—“make a muscle”
Mouth and jaw	“Open your mouth, as wide as you comfortably can”
Eyes and checks	“Close your eyes very tightly”
Shoulder blades	“Pull back your shoulders as much as possible so that your chest sticks out”
Shoulders	“Tens your shoulder muscles while you raise them as if to shrug them”
Neck	“Remain cautious when you flex the muscle. Stand straight and keep your eyes facing forward and then slowly bend your neck backward (look up at the ceiling)”
Chest and stomach	“Take a breath, deep enough to fill your lungs”
Hips and buttocks	“Tens your buttock muscles”
Upper leg	“Flex both your thighs”
Lower leg	“To prevent cramps, do this gently and be careful. To stretch your calf muscles, draw your toes toward yourself”
Foot	“Bend down your toes”

This table is taken from a recent study [27]

### Data analysis

The data analysis was performed using the SPSS software package (ver. 21), particularly descriptive (i.e., mean, SD, frequency, and percentage) and analytic tests, including Fisher's exact test, Chi-square test, independent-samples *t*-test, and paired-samples *t*-test. Kolmogorov–Smirnov test was utilized to check the normality of the data, considering the significance level of *p* > 0.05. The statistical analyst was blinded to the control and intervention groups.

## Results

### Individual characteristics

The nurses' mean age was 31.52 ± 6.36 (20–50 years), and the majority (60.9%) were between 20 and 30 years. 60.9% of the nurses were male, and 54.3% were single. The nurses' mean clinical work experience was 7.93 ± 4.95 (1–20 years), and most (39.1%) had clinical work experience of 1–5 years. Moreover, 82.6% of the nurses had a Bachelor's degree, and the majority (78.3%) were permanently employed. Furthermore, 41.3% of the nurses had rotating shifts. In the baseline, the mean score of stress and anxiety were 14.13 ± 2.56 and 13.06 ± 2.86, respectively. Of note, there were no significant differences between the experimental and control groups regarding individual characteristics (Table 2).

**Table 2:** Nurses' individual characteristics

**Groups**		**Experimental**	**Control**	**Test**	***p*** **-value**
**Variables**		**Mean (SD)**	**Mean (SD)**		
**Age, year**		31 (66.5)	32.04 (6.16)	^a^t = -0.552	0.584
**Number of children**		1.11 (0.78)	0.67 (0.77)	^a^t = 1.292	0.212
**Clinical work experience, year**		7.17 (4.57)	8.70 (5.30)	^a^t = -1.042	0.303
		n (%)	n (%)		
**Gender**	Male	15 (65.2)	13 (56.5)	Fisher's exact test	0.763
	Female	8 (34.8)	10 (43.5)		
**Marital status**	Single	14 (60.9)	11 (47.8)	Fisher's exact test	0.554
	Married	9 (39.1)	12 (52.2)		
**Education**	Bachelor's degree	20 (87)	18 (78.3)	Fisher's exact test	0.699
	Master's degree	3 (13)	5 (21.7)		
**Work shift**	Rotating	11 (47.8)	8 (34.8)	Fisher's exact test	0.573
	Morning	6 (26.1)	4 (17.4)		
	Evening	2 (8.7)	4 (17.4)		
	All day long	3 (13)	3 (13)		
	Night	1 (4.3)	4 (17.4)		

^a^Independent-samples *t*-test

### Stress and anxiety

The findings showed that there was no significant difference in the level of stress (*p* = 0.908) and anxiety (*p* = 0.233) of nurses in the two groups before the intervention, while there was a significant difference after the intervention (*p* < 0.001). So, nurses who experienced severe or extremely severe stress and anxiety were less in the experimental group than in the control group (Tables 3 and 4).

**Table 3:** Comparing nurses' stress levels in experimental and control groups before and after intervention

**Stage**	**Pre-test**						**Post-test**					
**Groups**	**Experimental**		**Control**		**Total**		**Experimental**		**Control**		**Total**	
**Stress level**	**n**	**%**	**n**	**%**	**n**	**%**	**n**	**%**	**n**	**%**	**n**	**%**
**Normal**	0	0	1	4.3	1	2.2	1	4.3	0	0	1	2.2
**Mild**	1	4.3	1	4.3	2	4.3	6	26.1	2	8.7	8	17.4
**Moderate**	5	21.7	3	13.0	8	17.4	11	47.8	3	13.0	14	30.4
**Severe**	14	60.9	14	60.9	28	60.9	5	21.7	16	69.6	21	45.7
**Extremely severe**	3	13	4	17.4	7	15.2	0	0	2	8.7	2	4.3
**Test, ** ***p*** **-value**	Fisher's exact test, X^2^ = 1.888, *p* = 0.908						Fisher's exact test, X^2^ = 14.744, *p* = 0.001

**Table 4:** Comparing nurses' anxiety levels in experimental and control groups before and after intervention

**Stage**	**Pre-test**						**Post-test**					
**Groups**	**Experimental**		**Control**		**Total**		**Experimental**		**Control**		**Total**	
**Anxiety level**	**n**	**%**	**n**	**%**	**n**	**%**	**n**	**%**	**n**	**%**	**n**	**%**
**Normal**	0	0	0	0	0	0	1	4.3	0	0	1	2.2
**Mild**	0	0	0	0	0	0	0	0	0	0	0	0
**Moderate**	1	4.3	1	4.3	2	4.3	2	8.7	0	0	2	4.3
**Severe**	3	13	0	0	3	6.5	10	43.5	1	4.3	11	23.9
**Extremely severe**	19	82.6	22	95.7	41	89.1	10	43.5	22	95.7	32	69.6
**Test, ** ***p*** **-value**	Fisher's exact test, X^2^ = 3.066, *p* = 0.233						Fisher's exact test, X^2^ = 14.635, *p* < 0.001					

The independent-samples *t*-test revealed that the mean score of stress (*p* = 0.571) and anxiety (*p* = 0.509) of the nurses in both experimental and control groups did not significantly differ at the pre-intervention stage, while the nurses in the experimental group experienced less stress (*p* < 0.001) and anxiety (*p* < 0.001) at the post-intervention. Based on Cohen’s method, the overall effect size of demonstration-based PMR on the nurses’ stress and anxiety was 1.47 and 1.61, respectively (Table 5).

**Table 5:** Comparing nurses' stress and anxiety scores in experimental and control groups before and after intervention

**Groups**		**Experimental**		**Control**		**Independent-samples ** ***t*** **-test**	
**Variables**		**Pre-test**	**Post-test**	**Pre-test**	**Post-test**	**Pre-test**	**Post-test**
**Stress**	**Mean** **(SD)**	13.91 (2.41)	10.95 (2.01)	14.34 (2.74)	14.17 (2.34)	t = -0.571 *p* = 0.571	t = -4.991 *p* < 0.001 Cohen's *d* = 1.47
	**Paired-samples ** ***t*** **-test**	t = 6.314 df = 22 *p* < 0.001		t = 1.164 df = 22 *p* = 0.257		*p* = 0.571	*p* < 0.001
**Anxiety**	**Mean** **(SD)**	13.34 (3.41)	9.47 (2.37)	12.78 (2.21)	12.91 (1.85)	t = -0.665 *p* = 0.509	t = -5.469 *p* < 0.001 Cohen's *d* = 1.61
	**Paired-samples ** ***t*** **-test**	t = 8.203 df = 22 *p* < 0.001		t = -0.720 df = 22 *p* = *0.479*			

*SD* standard deviation

As well, the paired-sample *t*-test showed that the levels of stress in the nurses in the experimental group reduced after the intervention (*p* < 0.001), while there was no significant difference in the control group (*p* = 0.257). Besides, the nurses' anxiety significantly declined at the post-intervention stage (*p* < 0.001), whereas no significant change was observed in the control group (*p* = 0.479) (Table 5).

This study did not have any harm or unintended effects on each group.

## Discussion

This study aimed to investigate the effectiveness of the demonstration-based PMR technique on the stress and anxiety of nurses caring for COVID-19 patients. The findings showed that in the baseline, prevalence rates of moderate to severe stress and anxiety nurses were 78.3% and 10.8%, respectively. Also, the prevalence rates of extremely severe stress and anxiety were 15.2% and 89.1%, respectively. A study showed that the prevalence rates of moderate to severe stress and anxiety in Iranian nurses in COVID‑19 care wards were 23.7% and 61.3%, respectively [5]. Also, Sheikhbardsiri et al. reported that the mean scores of stress and anxiety in nurses at educational hospitals during the COVID-19 pandemic were at a moderate level [4]. These findings emphasized that COVID-19 affected nurses’ mental health.

The mean score of stress and anxiety of all nurses were 14.13 ± 2.56 and 13.06 ± 2.86, respectively. Several previous studies reported that the mean score of stress was 9.62 ± 4.94 [4], 15.13 ± 4.76 [5], and 16.23 ± 9.25 [6] among nurses in Iran during the COVID-19 pandemic. Also, they reported that the mean score of anxiety was 10.32 ± 4.85 [4], 13.21 ± 4.90 [5], and 12.65 ± 9.52 [6]. The mean score of stress in Zakeri et al. [6] was higher than in other studies. They studied nurses during the first wave of the COVID-19 pandemic. It seems that at the beginning of the pandemic, nurses experienced higher stress and anxiety. Also, the nurses’ stress and anxiety in Sheikhbardsiri et al.’ study [4] that was conducted in Kerman were lower than in other cities in Iran. The study setting was one of the referral hospitals where patients with or suspected of COVID-19 were hospitalized during the entire pandemic. Therefore, there is a possibility that the nurses who worked in this hospital experienced higher stress and anxiety.

Findings showed that the given relaxation technique could significantly reduce the stress and anxiety scores in the experimental group. In this study, the overall effect size of demonstration-based PMR on the nurses’ stress and anxiety was 1.47 and 1.61, respectively. These effect sizes imply that this method had high effects on nurses’ stress and anxiety. In this regard, evidence showed that relaxation techniques effectively decreased stress and anxiety. According to Liu et al., PMR has a positive effect on reducing anxiety in patients with COVID-19 [26]. Özlü et al. mentioned that PMR can be considered a nonpharmacological method of reducing the anxiety of COVID‐19 patients [25]. Also, Toqan et al. confirmed that PMR has an effect in decreasing clinical experience anxiety among nursing students [27]. As well as, some researchers found that Benson's Relaxation Technique was an effective intervention strategy to relieve anxiety in intensive care unit nurses [30]. According to de Avila Silveira et al., relaxation techniques could effectively and significantly moderate stress in nurses [33]. Ozgundondu and Metin also reported that PMR and music could reduce the stress of nurses working in the critical care units, anesthesia, and internal wards of hospitals [32]. As stated by Jourabchi et al., Benson's Relaxation Technique was an effective strategy for decreasing work-related stress in midwives [34]. The evidence further showed that PMR alleviated stress and anxiety in nursing students [35] and some patients [32, 34]. The reason for the consistency between previous researches and the present study and the effectiveness of the intervention programs can be attributed to the nature of the relaxation technique as a complementary medicine founded on stress relief as a key element for moderation.

### Limitations

One of the limitations of the present study was the possibility of contamination bias. Thus, there were attempts to select two different hospitals for sampling to minimize it. Also, the nurses of the experimental group were requested not to share their information about PMR with the nurses of other hospitals until the end of the study. As well as the nursing population in Iran is mainly made up of women while most of the participants were men. However, the two groups were homogeneous in terms of gender. Also, as mentioned in the methodology section, the validity and reliability of the original version and the Persian version of the DASS-21 questionnaire were confirmed in the previous studies. However, it would have been better if it was re-examined in this study as well.

## Conclusion

In general, nurses working in intensive care units and the wards for COVID-19 patients undergo fatigue, work pressure, livelihood problems, and burnout caused by their profession, and observe pain, suffering, and death of patients, get infected with the virus, live with confirmed cases in their families, and experience high levels of stress and anxiety. Sometimes nurses are also ignored and burdened with much stress. High stress and anxiety of nurses can affect the quality of patient care.

The findings revealed that PMR was effective in reducing the stress and anxiety of the nurses caring for COVID-19 patients. In this view, it is recommended to implement this simple, affordable, and valid non-pharmacological intervention to help nurses cope with stress and anxiety and improve the quality of nursing services. Furthermore, it is suggested to assess the effects of this relaxation technique on other psychological complications caused by the care for COVID-19 patients in future research.

## Data Availability

The datasets analyzed in this study are available from the corresponding or first author upon reasonable request.

## Abbreviations

COVID-19: Coronavirus disease 2019
PMR: Progressive muscle relaxation
SD: Standard deviation
DASS-21: Depression Anxiety Stress Scale-21

## References

[1] Kim MPLY, Nguyen DT, Jones SL, Graviss EA, Phillips RA (2022). Health system strategy to safely provide surgical care during the Covid-19 Pandemic. NEJM Catal Innov Care Deliv.

[2] Farsi Z, Sajadi SA, Afaghi E, Fournier A, Aliyari S, Ahmadi Y (2021). Explaining the experiences of nursing administrators, educators, and students about education process in the COVID-19 pandemic: a qualitative study. BMC Nurs.

[3] Vajpeyee M, Tiwari S, Jain K, Modi P, Bhandari P, Monga G (2022). Yoga and music intervention to reduce depression, anxiety, and stress during COVID-19 outbreak on healthcare workers. Int J Soc Psychiatry.

[4] Sheikhbardsiri H, Doustmohammadi MM, Afshar PJ, Heidarijamebozorgi M, Khankeh H, Beyramijam M (2021). Anxiety, stress and depression levels among nurses of educational hospitals in Iran: Time of performing nursing care for suspected and confirmed COVID-19 patients. J Educ Health Promot.

[5] Sharifi A, Fallahi-Khoshknab M, Mohammadi S, Zeraati M, Jamshidi Z, Aghabeygi-Arani M (2022). Depression, anxiety, and stress among Iranian nurses in COVID-19 care wards. BMC Psychology.

[6] Zakeri MA, Rahiminezhad E, Salehi F, Ganjeh H, Dehghan M (2021). Burnout, Anxiety, Stress, and Depression Among Iranian Nurses: Before and During the First Wave of the COVID-19 Pandemic. Front Psychol.

[7] Pourgholam N, Zareiyan A, Farsi Z, Kurosh A. Exploring perceived stress from caring for coronavirus disease (COVID-19) patients in nurses: a qualitative study. J Res Nurs. 2022;00(0):1–13. 10.1177/17449871221131181 PMC9790853 36919106

[8] Teymouri F, Rajai N, Farsi Z, Pourmirzai M (2019). The effects of inhaling lavender fragrance on stress and anxiety during sheath takeout in patients after coronary angiography. J Med Plants.

[9] Nogee D, Tomassoni AJ (2020). Covid-19 and the N95 respirator shortage: Closing the gap. Infect Control Hosp Epidemiol.

[10] Shrestha GS (2020). COVID-19 Pandemic: Shortage of Personal Protective Equipment, Use of Improvised Surrogates, and the Safety of Health Care Workers. J Nepal Health Res Counc.

[11] Moayed MS, Mahmoudi H, Ebadi A, Salary MM, Danial Z (2015). Effect of education on stress of exposure to sharps among nurses in emergency and trauma care wards. Trauma Mon.

[12] Aghaei MH, Ebadi A, Aliakbari F, Vafadar Z (2020). The effectiveness of crisis management education based on inter-professional approach on military nurses’ ability to confront with crisis. J Mil Med.

[13] Fung OW, Loke AY, Lai CK (2008). Disaster preparedness among Hong Kong nurses. J Adv Nurs.

[14] MohamadzadehTabrizi Z, Mohammadzadeh F, DavariniaMotlaghQuchan A, Bahri N (2022). COVID-19 anxiety and quality of life among Iranian nurses. BMC Nurs.

[15] Ariapooran S, Ahadi B, Khezeli M (2022). Depression, anxiety, and suicidal ideation in nurses with and without symptoms of secondary traumatic stress during the COVID-19 outbreak. Arch Psychiatr Nurs.

[16] de Pinho LG, Sampaio F, Sequeira C, Teixeira L, Fonseca C, Lopes MJ (2021). Portuguese nurses' stress, anxiety, and depression reduction strategies during the COVID-19 outbreak. Int J Environ Res Public Health.

[17] Noorbala AA, Fathi Ashtiani A, Niknam MH, Emami Razavi SH, Ramezankhani A, Khayamzadeh M (2020). COVID-19 epidemic and mental health. Q J Health Promot.

[18] Heydari A, Abdollahi M (2021). Fear of Covid-19 in nurses: a concept analysis with a Walker-Avant approach. J Torbat Heydariyeh Univ Med Sci.

[19] Sharififardd F, Nazari N, Asayesh H, Ghanbari Afra L, Goudarzi Rad M, Shakeri M (2021). Evaluation of psychological disorders in nurses facing patients with Covid-19 in 2020. Qom Univ Med Sci J.

[20] Doo EY, Kim M, Lee S, Lee SY, Lee KY (2021). Influence of anxiety and resilience on depression among hospital nurses: a comparison of nurses working with confirmed and suspected patients in the COVID-19 and non-COVID-19 units. J Clin Nurs.

[21] Shareinia H, Khuniki F, BloochiBeydokhti T, Eydizeynabad A, Hosseini M (2018). Comparison between job stress among emergency department nurses with nurses of other departments. Q J Nurs Manag.

[22] Rahmatpour P, Karimi L, Rahimibashar F, Vahedian-Azimi A, Goharani R (2019). Effect of progressive muscle relaxation on the outcomes of multiple sclerosis disease: A systematic review and meta-analysis. J Nurs Educ.

[23] Ignatavicius DD, Workman ML, Heimgartner NM (2004). Medical-Surgical Nursing.

[24] Santos-Silva Ad, Bubols MN, Argimon IdL, Stagnaro O, Alminhana LO (2020). Benefits of relaxation techniques in the elderly: a systematic review. PSICO.

[25] Özlü İ, Öztürk Z, Karaman Özlü Z, Tekin E, Gür A (2021). The effects of progressive muscle relaxation exercises on the anxiety and sleep quality of patients with COVID-19: a randomized controlled study. Perspect Psychiatr Care.

[26] Liu K, Chen Y, Wu D, Lin R, Wang Z, Pan L (2020). Effects of progressive muscle relaxation on anxiety and sleep quality in patients with COVID-19. Complement Ther Clin Pract.

[27] Toqan D, Ayed A, Joudallah H, Amoudi M, Malak MZ, Thultheen I (2022). Effect of Progressive Muscle Relaxation Exercise on Anxiety Reduction Among Nursing Students During Their Initial Clinical Training: A Quasi-Experimental Study. Inquiry.

[28] Matourypour P, Ghaedi Heydari F, Bagheri I, Memarian R (2012). The effect of progressive muscle relaxation on the occupational stress of nurses in critical care units. Jorjani Biomed J.

[29] Teimouri F, Pishgooie SA, Malmir M, Rajai N (2019). The effect of Benson Relaxation on physiological criteria in patients undergoing coronary artery bypass graft surgery. Complement Med J.

[30] Najafi Ghezeljeh T, Sedghian H, Mohades AF (2016). Effect of Benson relaxation technique on anxiety in critical care nurses. Cardiovasc Nurs J.

[31] Taheri Gharagzlu T, Safavi M, Fesharaki M (2020). The effect of employee's humor training on depression, anxiety, and stress of the elderly residents in Tehran's nursing homes: a randomized clinical trial. Med Sci J.

[32] Ozgundondu B, Gok MZ (2019). Effects of progressive muscle relaxation combined with music on stress, fatigue, and coping styles among intensive care nurses. Intensive Crit Care Nurs.

[33] de Avila Silveira E dMBK, da Silva Grazziano E, de Oliveira Bringuete ME, Lima EdFA. Effect of progressive muscle relaxation on stress and workplace well‐being of hospital nurses. Enfermería Global. 2020;58:485–93.

[34] Jourabchi Z, Satari E, Mafi M, Ranjkesh F (2020). Effects of Benson's relaxation technique on occupational stress in midwives. Nursing.

[35] Zargarzadeh M, Shirazi M (2014). The effect of progressive muscle relaxation method on test anxiety in nursing students. Iran J Nurs Midwifery Res.

